# Repair of Cranial Bone Defects Using rhBMP2 and Submicron Particle of Biphasic Calcium Phosphate Ceramics with Through-Hole

**DOI:** 10.1155/2015/926291

**Published:** 2015-09-27

**Authors:** Byung-Chul Jeong, Hyuck Choi, Sung-Woong Hur, Jung-Woo Kim, Sin-Hye Oh, Hyun-Seung Kim, Soo-Chang Song, Keun-Bae Lee, Kwang-Bum Park, Jeong-Tae Koh

**Affiliations:** ^1^Research Center for Biomineralization Disorders and Department of Pharmacology and Dental Therapeutics, School of Dentistry, Chonnam National University, Gwangju 500-757, Republic of Korea; ^2^RIS Foundation for Advanced Biomaterials, Chonnam National University, Gwangju 500-757, Republic of Korea; ^3^Center for Biomaterials, Korea Institute of Science & Technology, Seoul 130-650, Republic of Korea; ^4^Department of Orthopedic Surgery, Chonnam National University Medical School and Hospital, Gwangju 501-757, Republic of Korea; ^5^Megagen Implant, Gyeongsan, Gyeongbuk 712-850, Republic of Korea

## Abstract

Recently a submicron particle of biphasic calcium phosphate ceramic (BCP) with through-hole (donut-shaped BCP (d-BCP)) was developed for improving the osteoconductivity. This study was performed to examine the usefulness of d-BCP for the delivery of osteoinductive rhBMP2 and the effectiveness on cranial bone regeneration. The d-BCP was soaked in rhBMP2 solution and then freeze-dried. Scanning electron microscope (SEM), energy dispersive spectroscopy (EDS), and Raman spectroscopy analyses confirmed that rhBMP2 was well delivered onto the d-BCP surface and the through-hole. The bioactivity of the rhBMP2/d-BCP composite was validated in MC3T3-E1 cells as an *in vitro* model and in critical-sized cranial defects in C57BL/6 mice. When freeze-dried d-BCPs with rhBMP2 were placed in transwell inserts and suspended above MC3T3-E1, alkaline phosphatase activity and osteoblast-specific gene expression were increased compared to non-rhBMP2-containing d-BCPs. For evaluating *in vivo* effectiveness, freeze-dried d-BCPs with or without rhBMP2 were implanted into critical-sized cranial defects. Microcomputed tomography and histologic analysis showed that rhBMP2-containing d-BCPs significantly enhanced cranial bone regeneration compared to non-rhBMP2-containing control. These results suggest that a combination of d-BCP and rhBMP2 can accelerate bone regeneration, and this could be used to develop therapeutic strategies in hard tissue healing.

## 1. Introduction

Bone defects caused by accidents, trauma, or delayed recovery from diseases can result in major clinical skeletal problems that require reconstruction to restore bone function [1, 2]. Autologous bone grafting is a widely used approach, especially in the regeneration of craniofacial bone defects [3]. However, autologous bone grafts have significant limitations, including often painful and limited access to the graft site, as well as morbidity to the donor site. Therefore, various synthetic biomaterials have been developed as bone substitutes to bone grafts, including bioactive ceramic, bioactive glasses, reinforced natural materials, and synthetic polymers [4].

Biphasic calcium phosphate ceramics (BCPs) are composed of two calcium phosphate phases: hydroxyapatite (HA) and beta-tricalcium phosphate (*β*-TCP) at a specific ratio, and they exhibit good biocompatibility and bone conduction performance [5]. However, pure BCP mainly acts as an osteoconductive substance with limited bone formation and relatively long regeneration time. Therefore, it is necessary to provide it with macro-/microporous structures for enhancing osteoconductivity or to combine the bioactive molecules such as bone morphogenetic proteins (BMPs) for improving osteoinductive properties.

Chemical composition, geometry, and macrostructural properties of BCP have been shown to play an important role in osteoconductivity. Porosity and pore size at both the macro- and microlevels are important morphological properties. Both influence bone healing and regeneration by allowing blood vessels to invade the material, supplying nutrients and oxygen and, thus, sustaining the cell metabolism inside the scaffold [6, 7]. Recently, a submicron particle of BCP ceramics (60 : 40 HA/*β*-TCP) with through-hole (donut-shaped BCP; d-BCP) was developed for improving the osteoconductivity, and their effectiveness on bone regeneration was determined in rabbit calvarial bone defects [6].

The recombinant human BMP 2 (rhBMP2) has been well characterized as a strong inducer of bone formation in a variety of conditions [8, 9]. A few animal and human studies have shown efficacious bone regeneration and healing with functional restoration after the implantation of rhBMP2 [10–13]. Therefore, combinations of bone substitutes and osteoinductive agents such as rhBMP2 have received increasing attention as potential bone graft substitutes [14]. Moreover, it has been reported that BMP-loaded HA/*β*-TCP ceramics greatly increase bone formation [15]. However, only a few studies have investigated the osteoinductivity of BMP-loaded BCP ceramics with porosity [13]. In the present study, we aimed to determine the usefulness of d-BCP for delivering rhBMP2 and the effectiveness on cranial bone regeneration in a well-documented animal model. We provide* in vitro* and* in vivo* evidence that a combination of rhBMP2 and d-BCP offered higher osteogenic and bone healing activities than that by d-BCP alone. Thus, the implantation of rhBMP2/d-BCP could provide a significant approach to clinical bone regeneration and reconstruction.

## 2. Materials and Methods

### 2.1. Recombinant Proteins and Materials

rhBMP2 was purchased from Cowellmedi (Seoul, Korea). Submicroporous biphasic calcium phosphate ceramics with through-hole (d-BCP; Bone Plus), a mixture of HA/*β*-TCP (60 : 40), was kindly supplied by Megagen Implant Co. (Gyeongsan, Korea).

### 2.2. Delivery of rhBMP2 onto the d-BCP

Ten mg of d-BCP was soaked into 1 mL of rhBMP2 solution (5 *μ*g/mL) and then freeze-dried. Successful delivery of rhBMP2 onto the d-BCP surface or through-hole was verified by morphological and compositional analyses. The surface morphology of the freeze-dried d-BCP with rhBMP2 was observed using field emission scanning electron microscopy (FE-SEM, Hitachi, Tokyo, Japan; Korea Basic Science Institute, Gwangju Center). The surfaces were sputter-coated with platinum and voltages ranging from 5 to 15 kV were used. In addition, compositional analysis using energy dispersive spectroscopy (EDS, Bruker AXS, Karlsruhe, Germany; Korea Basic Science Institute) attached to SEM was carried out. Micro-Raman spectrum was also recorded for d-BCP with rhBMP2 (rhBMP2/d-BCP) in the spectral range of 100–4000 cm^−1^ by using a micro-Raman spectrometer (InVia Reflex UV Raman microscope, Renishaw, UK; Korea Basic Science Institute). A He–Ne laser at 15 mW was used with an excitation wavelength of 633 nm and a resolution of 1.2 cm^−1^.

### 2.3. Cell Culture

MC3T3-E1 preosteoblasts were seeded at a density of 2.5 × 10^4^ cells/cm^2^ in 6-well Transwell plates (SPL Inc., Seoul, Korea) and grown with *α*-minimal essential medium (*α*-MEM; Invitrogen, Carlsbad, CA, USA), supplemented with 10% fetal bovine serum (FBS; Gibco, BRL, USA) and 1% penicillin/streptomycin (Invitrogen, Carlsbad, CA, USA), in a humidified atmosphere of 5% CO2 at 37°C. For the transwell cultivation, freeze-dried d-BCPs with or without rhBMP2 were placed in transwell inserts and suspended above the cell cultures using three wells per sample group, allowing for release of the factor from the matrix without direct cell contact. After 72 h, the lower cells were harvested for further analysis.

### 2.4. Total RNA Extraction and RT-PCR

Total RNA was isolated from the cultured cells using TRIzol reagent (Invitrogen) according to the manufacturer's instructions. To amplify the transcripts of osteoblast-specific genes, cDNA was synthesized from 1 *μ*g of total RNA using random primers and SuperScript II reverse transcriptase (200 units; Invitrogen), and then polymerase chain reaction was performed. The reaction consisted of an initial denaturation step at 94°C for 1 min, followed by a three-stage cycle: denaturation at 94°C for 30 s, annealing at a temperature optimized for each primer pair for 30 s, and extension at 72°C for 30 s. After the requisite number of cycles, the reactions underwent a final extension at 72°C for 5 min. Annealing temperatures, number of cycles, and primer sequences for alkaline phosphatase (ALP), osteocalcin (OC), osterix (Osx), and *β*-actin are as follows: ALP (55°C, 25 cycles), (F) 5′-TACATTCCCCATGTGATGGC-3′ and (R) 5′-ACCTCTCCCTTGAGTGTGGG-3′; OC (55°C, 25 cycles), (F) 5′-CTCCTGAGTCTGACAAAGCCTT-3′ and (R) 5′-GCTGTGACATCCATTACTTGC-3′; Osx (55°C, 25 cycles), (F) 5′-TGAGGAAGAAGCCCATTCAC-3′ and (R) 5′-ACTTCTTCTCCCGGGTGTG-3′; *β*-actin (55°C, 25 cycles), (F) 5′-TGGATGGCTACGTACATGGCTGGG-3′ and (R) 5′-TTCTTTGCAGCTCCTTCGTTGCCG-3′. The amplified PCR products were electrophoresed on a 1.5% agarose gel and visualized by RedSafe Nucleic Acid Staining solution (Intron Biotechnology, Sungnam, Korea) using the i-MAX gel image analysis system (CoreBioSystem, Seoul, Korea).

### 2.5. Alkaline Phosphatase (ALP) Staining

To examine effects of rhBMP2/d-BCP on bioactivity of bone-forming osteoblasts, ALP staining was performed in MC3T3-E1. Cells were fixed with 70% ethanol, rinsed three times with deionized water, and then treated for 15 min with a 5-bromo-4-chloro-3-indolyl phosphate/nitro blue tetrazolium solution (Sigma Aldrich, St. Louis, MO, USA). For quantitative analysis, the stains were extracted with 10% (w/v) cetylpyridinium chloride in 10 mM sodium phosphate (pH 7.0) for 15 min, and absorbance was measured with microplate reader (Multiskan GO; Thermo Scientific, Waltham, USA) at 540 nm.

### 2.6. Animal Preparations

All animal studies were reviewed and approved by the Animal Ethics Committee of Chonnam National University (number CNU-IACUC-YB-2014-35). Six-week-aged male C57BL/6 mice were obtained from Daehan Biolink (Chungbuk, Korea), and 10 mice per group were randomly assigned. Animals were anesthetized by intraperitoneal injection of a mixture of Zoletil (30 mg/kg; Virbac Lab, Carros, France) and Rompun (10 mg/kg; Bayer Korea Ltd., Seoul, Korea). A sagittal incision was made on the scalp and the calvarium was exposed. A critical-sized bone defect was created by using a 5 mm inner diameter trephine bur (Fine Science Tools, Foster City, CA, USA) under low speed drilling and cool saline irrigation. The defects were filled with d-BCP (10 mg) or rhBMP2/d-BCP composites (5 or 10 *μ*g of rhBMP2 with 10 mg of d-BCP) according to group. In the control group, the defects were unfilled. The animals were sacrificed 2 and 8 weeks after surgery by CO2 asphyxiation. The crania were carefully removed and fixed for 24 h in 10% neutral buffered formalin solution and then transferred into 70% ethyl alcohol for storage.

### 2.7. Soft X-Ray and Microcomputed Tomography (Micro-CT) Scanning

The whole body and the isolated crania from each mouse were radiographed by 2-dimensional radiographic apparatus (Hitex Ltd., Osaka, Japan) using diagnostic X-ray film (X-OMAT V, Kodak, Rochester, NY, USA) under the following conditions 35 kVp and 400 *μ*A for 45 s. For a 3-dimensional analysis, each specimen was scanned by micro-CT (Skyscan 1172; Skyscan, Aartselaar, Belgium) in cone-beam acquisition mode. The X-ray source was set at 50 kV and 200 *μ*A with a 0.5 mm aluminum filter at 17.09 *μ*m resolution. The exposure time was 1.2 s. 449 projections were acquired over an angular range of 180° (angular step; 0.4°). The image slices were reconstructed by using the NRecon program (version 1.6.2.0, Skyscan, Aartselaar, Belgium) and bone volume and thickness were measured using the CT-Analyzer program (version 1.10.0.5, Skyscan, Aartselaar, Belgium). 3D surface rendering images were obtained by using the Mimics software 14.0 (Materialise NV, Leuven, Belgium).

### 2.8. Histological Analysis

All specimens were decalcified in a rapid decalcifying solution (Calci-Clear Rapid, National Diagnostics, Atlanta, USA) for 10 days and then embedded in paraffin and cut into 7 *μ*m thick serial slices. The sections were deparaffinized in xylene at room temperature for 20 min and then rehydrated through a graded series of alcohols. The sections were then stained with hematoxylin and eosin (H&E). The H&E-stained sections from each group were then examined under a light microscope (Leica, Wetzlar, Germany) to evaluate new bone formation.

### 2.9. Statistical Analysis

Statistical analysis was performed using one-way analysis of variance (ANOVA) and Duncan's multiple comparisons using the Graph Pad Prism 4 for Windows statistical software package (Graph Pad Software Inc., La Jolla, CA, USA). All the data presented are expressed as the mean ± SEM from three independent measurements. A *p* < 0.05 was considered statistically significant.

## 3. Results

### 3.1. Surface Morphology and Compositional Analyses of rhBMP2/d-BCP

**Figure 1:**
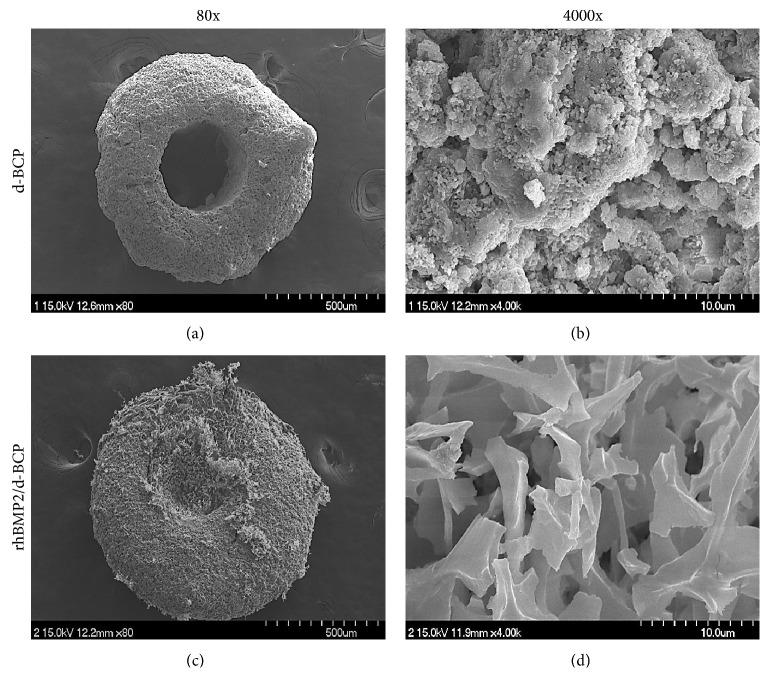
Morphology of the donut shape of microporous biphasic calcium phosphate ceramics (d-BCP) in the presence or absence of rhBMP2 (recombinant human bone morphogenetic proteins 2) was analyzed with SEM. d-BCP (10 mg) was soaked into rhBMP2 solution (5 *μ*g/mL) and then freeze-dried. (a, b) d-BCP, (c, d) rhBMP2/d-BCP, and (b, d) high magnification image of the d-BCP with and without rhBMP2.

The osteoconductive d-BCP was soaked into the osteoinductive rhBMP2 solution and then freeze-dried. Morphological analysis with FE-SEM showed that the d-BCPs were 500 and 700 *μ*m size of spherical particles with macro-/microinterconnected pore structures and a central through-hole (Figures 1(a) and 1(b)). The freeze-dried d-BCP with rhBMP2 solution (rhBMP2/d-BCP) had an irregular or closed spherical morphology with a relatively rough surface compared to d-BCP, indicating good packaging of rhBMP2 in the central through-hole of d-BCP (Figures 1(c) and 1(d)).

**Figure 2:**
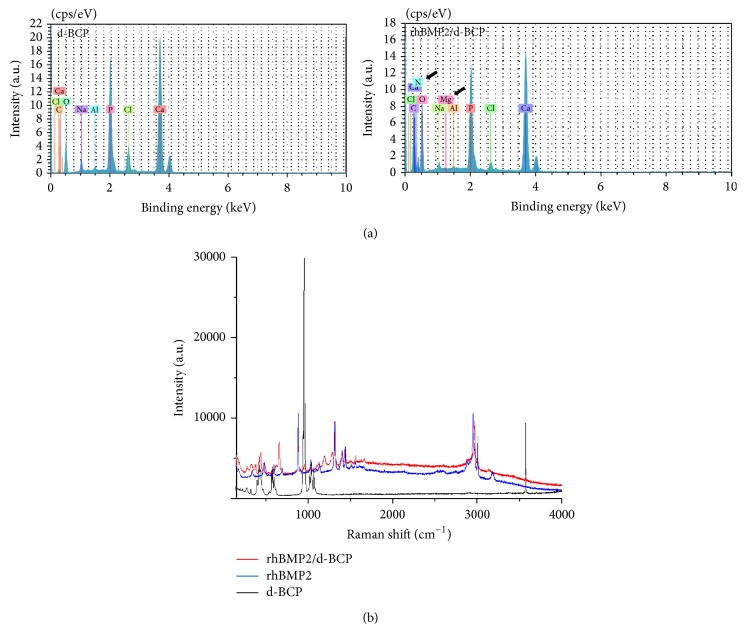
Energy dispersive spectra (EDS) and Raman spectroscopy were used to identify the chemical composition of the d-BCP with or without rhBMP2. (a) EDS profiles of d-BCP with and without rhBMP2, (b) Raman spectra of d-BCP (bottom), rhBMP2 protein (center), and rhBMP2/d-BCP (top) samples in the range of 100–4000 cm^−1^.

In the compositional analysis of EDS, an N and Mg layer was observed on the surface of the rhBMP2/d-BCP but not on that of d-BCP itself (Figure 2(a)). Raman spectroscopy analysis revealed that lots of peaks of rhBMP2/d-BCP are accorded with those of rhBMP2 itself, indicating that rhBMP2 can be transferred on the surface or through-hole of d-BCP (Figure 2(b)).

### 3.2. *In Vitro* Osteogenic Differentiation by the rhBMP2/d-BCP

**Figure 3:**
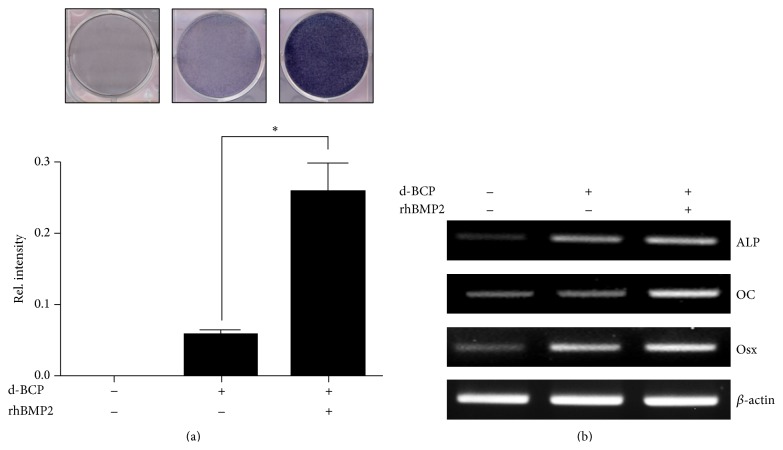
The effect of rhBMP2/d-BCP on osteogenic differentiation in MC3T3-E1 cells. Cells were maintained for 3 days in growth medium with d-BCP (1 mg) or rhBMP2 (0.5 *μ*g)/d-BCP (1 mg). (a) The cells were subjected to ALP staining. (b) Total RNA was isolated and expression of osteoblast-specific genes was analyzed by RT-PCR. *β*-actin was used as a loading control. ALP: alkaline phosphatase. OC: osteocalcin. Osx: osterix. ^*∗*^ *p* < 0.05 compared to the indicated group. Representative data are shown. *n* = 3.

ALP activity is widely used as a marker for the early differentiation of osteoblasts [16]. To examine the effects of the rhBMP2/d-BCP on osteogenic differentiation* in vitro*, preosteoblast MC3T3-E1 cells were maintained for 3 days in a transwell system, containing either d-BCP or rhBMP2/d-BCP, and then ALP staining was performed. ALP activity significantly increased in models with the rhBMP2/d-BCP compared to those with the control d-BCP group (Figure 3(a)). The expression levels of osteoblast-specific genes (such as ALP, OC) and Osx were also significantly higher in models with the rhBMP2/d-BCP than in the control models with only d-BCP (Figure 3(b)).

### 3.3. *In Vivo* Bone Formation by the rhBMP2/d-BCP

**Figure 4:**
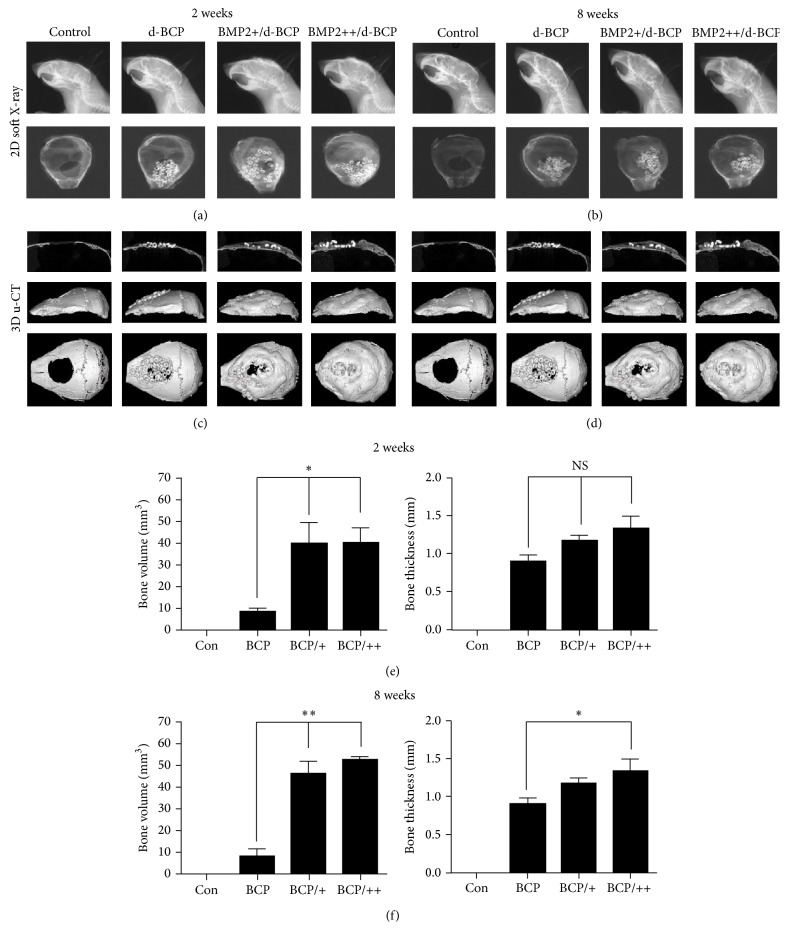
Effects of rhBMP2/d-BCP on bone repair of calvarial defects in mice. d-BCP (10 mg) or rhBMP2 (5 or 10 *μ*g)/d-BCP (10 mg) were implanted into a 5 mm inner diameter cranial defect. Control group was left without any implantation. The mice were harvested at 2 and 8 weeks after implantation, and 2D soft X-ray (a, b) and 3D microcomputed tomography (c, d) analyses were performed. Volume and thickness of regenerative bone were measured using micro-CT apparatus and micro-CT-Analyzer program (e, f). ^*∗*^ *p* < 0.05 and ^*∗∗*^ *p* < 0.01 compared to the indicated group. Representative data are shown. *n* = 5.

To evaluate bone formation by the rhBMP2/d-BCP* in vivo*, we implanted either d-BCP or rhBMP2/d-BCP (5 or 10 *μ*g) into a 5 mm inner diameter cranial defect, which was created in the central part of the mouse cranial bone. To visualize the regions of bone healing, soft X-ray and micro-CT analysis were performed 2 and 8 weeks after surgery. X-ray analysis revealed that the control group without the scaffold showed round and radiolucent cranial defects for up to 8 weeks. In the group with d-BCP implant, both d-BCP particles and a radio-opaque shadow around the defects were detected at 2 weeks. After 8 weeks, the radio-opaque shadow decreased and new bone was detected in the spaces between the particles. However, the cranial defect had not completely healed (Figures 4(a) and 4(b)). In the groups with rhBMP2/d-BCP implants, d-BCP particles and a rounded radio-opaque shadow were also shown. However, the cranial defect had healed more effectively in this group. The 3-dimensional analyses of defects using micro-CT scanning showed that the group with rhBMP2/d-BCP implants had substantial platelike bone structure, which was visible in the center of the cranial defect, and that this group had a higher capacity for healing in the peripheral area surrounding the defect, compared to the group with d-BCP implants (Figures 4(c) and 4(d)). Volumetric analysis using micro-CT also demonstrated that bone volume in the defects with rhBMP2/d-BCP implant is greater than that with d-BCP implant alone. The thickness of newly regenerated bone was also significantly higher in the rhBMP2/d-BCP groups than in the d-BCP group. When compared to the negative control, the group with d-BCP implants alone also exhibited a significant increase in both bone volume and bone thickness (Figures 4(e) and 4(f)).

### 3.4. Histological Analysis

**Figure 5:**
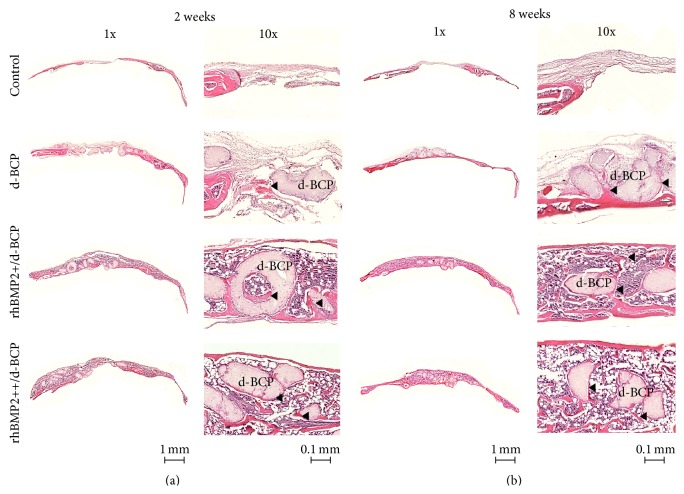
Histological analysis of rhBMP2/d-BCP induced bone regeneration in calvarial defects of mice. All specimens used for radiographic analyses (Figure 4) were formalin-fixed, paraffin-embedded, and then cut into 7 *μ*m thick sections. The sections were then stained with hematoxylin and eosin (H&E). Micrographs are shown at ×1 and ×10 magnifications.

Histological analysis was performed using H&E stained sections at 2 and 8 weeks after implantation, in order to qualitatively evaluate new bone formation. In the control group, no mineralized bone was observed in the empty cranial defect and instead the thin fibrous tissue coverage was seen. In the group with d-BCP implants, only a small amount of newly formed bone was found in the limited peripheral region of d-BCP particles at 8 weeks (Figure 5). However, the group with the rhBMP2/d-BCP implants showed greater amounts of bone regeneration with normal bone-like structure compared to the group with the d-BCP implants. In the rhBMP2/d-BCP group, the newly regenerated bone almost covered the outer surface as well as inner through-hole surface of d-BCP particles and was observed in the interparticular space (Figures 5(a) and 5(b)), suggesting that the regeneration may be affected by BMP2 adsorption on d-BCP.

## 4. Discussion

In this study, we investigated whether donut shape of BCP is useful for delivering osteogenic rhBMP2 and the delivery can synergically enhance bone regeneration in cranial defects of mice. Our results showed that BMP2 can be adsorbed on the microspore surface of BCP and plugged the central through-hole by freeze-drying and that the rhBMP2-adsorbed d-BCP (rhBMP2/d-BCP) enhanced* in vitro* osteoblast differentiation and* in vivo* bone formation, compared to d-BCP alone.

BCP integrates the excellent mechanical properties of less resorbable HA with faster resorbable *β*-TCP, and a HA/*β*-TCP ratio of 60 : 40 has been reported as the optimal composition for synthetic bone in previous animal studies [17, 18]. Previously the donut shape of BCP (d-BCP; HA/*β*-TCP ratio of 60 : 40), which is made of submicron-sized grains with 300–400 *μ*m central pore and 20–60 *μ*m micropores on surface, was developed as a bone substitute and characterized to have osteoconductivity [6]. In the present study, we consistently observed that implantation of d-BCP alone partly induced cranial bone regeneration in mice. We still consider that the response might come from the increase of osteoconductivity of d-BCP due to the surface characteristics with interconnected microporosity and through-hole, allowing some space for migrating osteoblasts and endothelial cells and contributing to vascularization and bone ingrowth.

So far, there are lots of trials to deliver osteoinductive BMP2 onto BCP particles and the enhancement of bone regeneration by them has been introduced with the limited functional evaluation [1]. This study was also undertaken with a hypothesis that d-BCP will be a more powerful bone substitute if osteoinductive substances are delivered into the macro-/micropore structures and the central through-hole of d-BCP. When d-BCP was soaked with rhBMP2 solution and freeze-dried in the present study, adsorption of rhBMP2 on the surface of d-BCP and through-hole was identified by morphological and compositional analyses such as SEM, EDS, and Raman spectrum. SEM images showed that the postlyophilized remnants roughly covered the outer surface of d-BCP particles and also plugged a central through-hole. In the EDS results, nitrogen and magnesium layers were observed in the surface of rhBMP2/d-BCP, not in that of d-BCP only [19]. Because nitrogen is a component of amino acid and magnesium is one of the protein binding inorganics [20], we can assume that postlyophilized remnants on the surface of rhBMP2/d-BCP might be BMP2 protein. Raman spectra analysis showed that lots of peaks of rhBMP2/d-BCP are accorded with those of rhBMP2 itself, indicating that rhBMP2 can be transferred on the surface or through-hole of d-BCP. Because d-BCP has a 300–400 *μ*m of central through-hole unlike previous plain particle type of BCPs, it has an advantage to deliver more rhBMP2 and to enhance bone regeneration.

BMP2 is the most potent osteoinductive growth factor to stimulate the development of endogenous bones or repair of damaged bones [21, 22]. In addition, BMP2 stimulates osteoblastic differentiation from mesenchymal stem cells or progenitor cells with the increases in osteoblast-specific gene expressions, including alkaline phosphatase enzyme, bone matrix proteins, and transcription factors [23, 24].

In this study, we further examined whether the adsorbed rhBMP2 on d-BCP surface still has such a stimulatory effect on osteoblast differentiation and bone regeneration. Our results of* in vitro* culture experiments showed that MC3T3E1 preosteoblasts with rhBMP2/d-BCP produced more increases in ALP enzyme activity, gene expression of ALP, bone matrix protein osteocalcin, and transcription factor osterix, compared with d-BCP alone. The results indicate that the rhBMP2 on d-BCP surface still has biological activity regardless of lyophilized process and adsorption on d-BCP surface.

Our* in vivo* study confirmed the rhBMP2/d-BCP effects on bone regeneration; rhBMP2/d-BCP implants induced greater bone regeneration, compared to d-BCP alone, in the critical-sized calvarial defects in mice. In the radiographic analysis, d-BCP alone also induced bone repair of calvarial defects as in a previous report [6]; however, the defects were not completely covered with new bones even at 8 weeks after implantation. On the other hand, rhBMP2/d-BCP implant significantly enhanced the bone repair with increases in bone volume and thickness in the defects, and the d-BCP with 10 *μ*g of rhBMP2 produced the completed healing even at 2 weeks after implantation; the defect was fully covered with new regenerated bone. However, the volume of new bone by the combination at 8 weeks was not increased, compared to that at 2 weeks. These indicate that rhBMP2 can initially burst from the rhBMP2/d-BCP complex to be inactive after 8 weeks or d-BCP itself may be improper to slowly release rhBMP2. For more efficient regeneration for long time, a sustained releasing system for rhBMP2 has to be added to the combination.

Histology results consistently revealed that d-BCP alone also produced new bone formation; however, that new bone was observed in the limited surface of d-BCP. On the other hand, the rhBMP2/d-BCP implant elicited to greater amounts of bone regeneration than the d-BCP implants; the newly regenerated bones almost covered the outer surface as well as inner through-hole surface of d-BCP particles and even were observed in interspace between d-BCP particles. The bone-forming pattern appears to be closely related to the rhBMP2 adsorption on d-BCP particles, when we consider the putative localization of rhBMP2 and osteoinductive activity. These consistently suggest that the adsorbed rhBMP2 has a stimulatory effect on* in vivo* bone regeneration. However, the different concentrations (5 *μ*g or 10 *μ*g) of rhBMP2 appear to have no effect on the maturity of new bone, indicating that the doses of rhBMP2 might not be enough to produce the matured bone in the presence of d-BCP in mice. To develop an optimal combination system using rhBMP2 and d-BCP for cranial bone regeneration, further studies are still needed including a sustained release strategy for long-term effects of rhBMP2, degradation behavior of d-BCP, appropriate concentration of rhBMP2, and so forth.

## 5. Conclusions

This study showed that donut shape of BCP (d-BCP) can deliver rhBMP2 through the hole with freeze-drying and that the rhBMP2/d-BCP can stimulate* in vivo* bone regeneration as well as* in vitro* osteogenic differentiation and mineralization. This rhBMP2 delivery system can be used to develop therapeutic strategies in bone regeneration and defect healing.
